# Anoikis-related CTNND1 is associated with immuno-suppressive tumor microenvironment and predicts unfavorable immunotherapeutic outcome in non-small cell lung cancer

**DOI:** 10.7150/jca.89542

**Published:** 2024-01-01

**Authors:** Yingchen Ni, Mengna Jiang, Yixuan Wu, Pei Xiao, Anqi Wu, Weiyi Xia, Can Tang, Xu Yang, Kai Tian, Hong Chen, Rongrong Huang

**Affiliations:** 1 Affiliated Hospital of Nantong University, Medical School of Nantong University, Nantong, 226001, China.; 2 Department of Occupational Medicine and Environmental Toxicology, Nantong Key Laboratory of Environmental Toxicology, School of Public Health, Nantong University, Nantong 226019, China.; 3 Center for Non-Communicable Disease Management, Beijing Children's Hospital, Capital Medical University, National Center for Children's Health, Beijing, China.; 4 Department of Respiratory Medicine, Nantong Fourth People's Hospital, Nantong, 226000, China.; Yingchen Ni, Mengna Jiang, and Yixuan Wu contributed equally.

**Keywords:** CTNND1, NSCLC, biomarker, immunotherapy, anoikis

## Abstract

**Background:** Immunotherapy has greatly changed the treatment of advanced non-small cell lung cancer (NSCLC). Anoikis is a programmed cell death process associated with cancer. However, the correlation between anoikis-related genes and the tumor microenvironment (TME) features and immunotherapeutic outcome in NSCLC has not been fully explored.

**Methods:** The bulk and single-cell transcriptome data of NSCLC were downloaded from TCGA and GEO databases. The distribution of anoikis-related genes on different cell types at the single-cell level was analyzed, and these genes specifically expressed by tumor cells and immunotherapy-related were further extracted. Next, the candidate gene CTNND1 was identified and its correlations with the TME features and immunotherapeutic outcome in NSCLC were explored in multiple public cohorts. Finally, an in-house cohort was used to determine the CTNND1 expression and immuno-correlation in NSCLC.

**Results:** At single-cell atlas, we found that anoikis-related genes expressed specifically in tumor cells of NSCLC. By intersecting anoikis-related genes, immunotherapy-associated genes, and the genes expressed in tumor cells, we obtained a special biomarker CTNND1. In addition, cell-cell communication analysis revealed that CTNND1+ tumor cells communicated with immune subpopulations frequently. Moreover, we found that high expression of CTNND1 was related to immuno-suppressive status of NSCLC. The expression of CTNND1 and its immuno-correlation were also validated, and the results showed that CTNND1 was highly expressed in NSCLC tissues and tumors with high CTNND1 expression accompanied with low CD8+ T cells infiltration.

**Conclusions:** Overall, our study reported that CTNND1 can be considered as a novel biomarker for the predication of immunotherapeutic responses and a potential target for NSCLC therapy.

## Introduction

Early detection of lung cancer is challenging, leading to decreased survival rates. Lung cancer is the primary cause of cancer mortality globally, and it has the highest incidence rate in China 1. Non-small cell lung cancer (NSCLC) represents the majority (80-90%) of all lung cancers, with lung adenocarcinoma and lung squamous cell carcinoma being the most common subtypes 2. Smoking, including primary and second-hand exposure, accounts for over 80% of lung cancer cases. Unfortunately, most patients have already progressed to advanced stages at the time of diagnosis. Platinum chemotherapy is the standard treatment method, however, the response to these drugs is typically not significant, and the disease progresses rapidly 3, 4.

Recently, immunotherapy has become an exciting treatment option for patients without driving mutations, and it has greatly changed the treatment of advanced NSCLC. Cancer immunotherapy involves the application of strategies aimed at enhancing the body's immune system to recognize and eradicate tumor cells 5, 6. Under normal circumstances, it is generally believed that the immune cells in the tumor microenvironment (TME) can distinguish and eliminate cancer cells, which is called immune surveillance 7-10. However, the advantages in cell and tumor can regulate the immune system to evade immune surveillance by recruiting immunosuppressive cells and acquiring immunosuppressive 11. In the process of tumor progression, even though antigen specific T cells can stimulate adaptive immune response, immune selection will also produce tumor cell variants, which will make tumor cells lose major histocompatibility complex (MHC) class I and II antigens expression on their surface as an immune escape mechanism 12. Thus, although immune checkpoint blockade (ICB) has significant survival benefits for a proportion of advanced NSCLC patients, a large number of patients still exhibit primary drug resistance 13, 14.

Normal cells will gather together and adhere to the cell basement membrane (ECM), surviving through mutual material and signal transduction. Once they lose contact with the ECM, the cells undergo programmed death or apoptosis. This form of cell death was first named anoikis in 1994 15, 16. The triggering of anoikis mainly occurs through the interaction of two apoptosis pathways, including the activation of cellular death receptors or through the mitochondria-driven activation of caspase-3 17-19. Resistance to anoikis is a characteristic of tumor metastasis, which allows tumor cells to spread to distant organs through the circulatory system. After detachment from the extracellular matrix and intercellular contact, tumor cells survive by resisting apoptosis through paracrine and autocrine mechanisms, and regain the ability to adhere, spread, and invade 20. Some tumor cells can resist anoikis through oxidative stress. For example, ROS is a key molecule that triggers cell survival signals. ROS activates downstream signaling pathways of ERK and Akt through oxidative activation of tyrosine kinases, leading to Bim degradation and preventing anoikis 21.

In this study, we first investigated the expression of anoikis-related genes in NSCLC, by analyzing the distribution of various genes on different cell types at the single-cell level, and identified the gene set specifically expressed by tumor cells. Secondly, we screened the gene set related to the efficacy of immunotherapy. Based on the above research, we found that the CTNND1 expression can guide immunotherapy as a powerful prognostic marker for NSCLC. Finally, we explored the relationship between the expression of CTNND1 and the TME features, as well as immune regulation biological processes. Exploring the expression patterns of genes related to anoikis not only expands our understanding of the invasiveness of NSCLC, but also helps to develop more personalized and precise treatment strategies.

## Materials and methods

### Dataset acquisition

The normalized RNA-sequencing profile and clinical annotations of patients in TCGA-NSCLC cohort were downloaded from the UCSC Xena website (https://xenabrowser.net/datapages/). GSE42127 22, the validation cohort, were downloaded from the Gene Expression Omnibus (GEO) portal (https://www.ncbi.nlm.nih.gov/geo/). Furthermore, the normalized gene expression profile of NSCLC clinical cohorts with anti-PD-1 therapy (GSE126044 23 and GSE135222 24) were also obtained from the GEO database. Also, immunotherapy cohorts of other epithelial tumors, including breast cancer (breast cancer: GSE173839 25), melanoma (PRJEB23709 26), and gastric carcinoma (PRJEB25780 27), were also obtained from public database. Samples with overall survival (OS) above zero-day were included in this research. For the immunotherapy cohort, diagnostic patients who received immunotherapy were selected for further analysis. In addition, the the anoikis-related genes were obtained from the Harmonizome portals 28 (https://maayanlab.cloud/Harmonizome/, accessed on 12 October 2022). The information of datasets used in this study was listed in Table S1.

### Single-cell RNA sequencing datasets analysis

The single-cell RNA sequencing datasets of 12 patients with NSCLC from the GSE150660 29, GSE127465 30 and GSE117570 31 were downloaded. All additional analyses were performed using the Seurat (4.0.4, http://satijalab.org/seurat/) R toolkit 32, including quality control and all subsequent analyses. To eliminate the influence of abnormal cells and technical background noise on downstream analysis, cells were reserved if the expression of mitochondrial genes was greater than 10% or with detected genes less than 200 or greater than 5,000. Finally, a total of 47,359 cells were used for further analysis.

**Figure 1:**
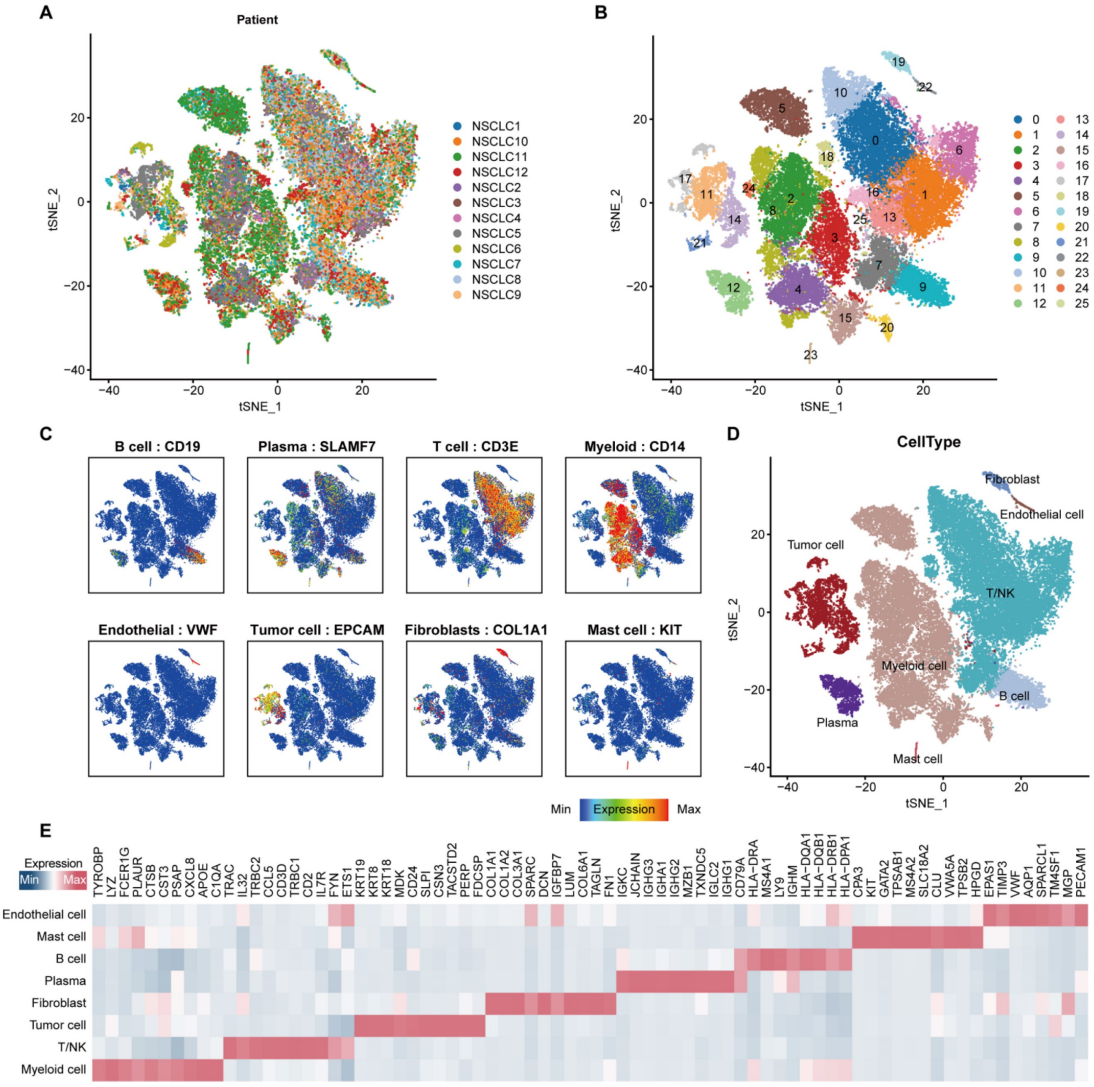
** Integrated scRNA-seq analysis of tumor tissues from NSCLC patients.** (A) t-SNE visualization of 47,359 single cells passed quality controls, colored by 12 NSCLC patients. (B) The unsupervised clustering of 47,359 cells. (C) Expression levels of known markers for specific cell types overlaid on the t-SNE representation. (D) t-SNE visualization of cell types annotated by classical gene markers. (E) Heatmap for gene expression levels of top ten cell-type-specific genes. The data in Figure 1 were downloaded from the GSE150660, the GSE127465, and the GSE117570 datasets.

In order to minimize the technical batch effects among individuals and experiments, we used the “RunHarmony” function in R package harmony 33 to integrate 47,359 cells from 12 NSCLC patients. The top 4,000 variable genes were used for principal component analysis (PCA) to reduce dimensionality. The dimensionality of the scaled integrated data matrix was further reduced to two-dimensional space based on the first 30 principal components (PCs) and visualized by t-Distributed Stochastic Neighbor Embedding (t-SNE). The cell clusters were identified based on a shared nearest neighbor (SNN) modularity optimization-based clustering algorithm with a resolution of 1, and all cells were divided into 26 clusters (Figure 1B). In order to recognize the types of these cells, some known markers, such as VWF for endothelial cells, EPCAM for epithelial cells, DCN for fibroblasts, CD3D for T cells, were used to verify the annotation of cell types (Figure 1C).

To validate the results found from the integrated scRNA-seq datasets of the GSE150660 29, GSE127465 30 and GSE117570 31, another scRNA-seq datasets (GSE131907 34) including 11 NSCLC patients were downloaded from the GEO website. Cell filtration, integration, and annotation were followed the criteria used above.

### Identification of differential expressed genes (DEG)

To identify the tumor cell-specific genes, “FindAllMarkers” function was performed. Genes with the |fold-change (FC)| ≥ 1.2, pct.1 ≥ 0.4, pct.2 ≤ 0.1, and adjusted P-values < 0.05 were identified as the tumor cell-specific genes.

In order to recognized the immunotherapeutic-related genes, the the R package “limma” 35 was used to perform the differential expression analysis between the NSCLC patients who received (R) and not received remission (NR) after immunotherapy in the GSE126044 cohort. Genes with the | FC| ≥ 1.5 and adjusted P-values < 0.05 were identified as the immunotherapeutic-related genes.

In order to identify the DEGs for CTNND1-high and low groups respectively, the R package “limma” 35 was used to perform the differential expression analysis. Genes with the FC ≥ 1.5 and adjusted P-values < 0.05 were defined as up-regulated genes for CTNND1-high group, while genes with the FC ≤ -1.5 and adjusted P-values < 0.05 were recognized as up-regulated for CTNND1-low group.

### Assessment of immunological characteristics of the TME

The associations between CTNND1 and the immunological features of the TME was evaluated 36. In order to assess the immunological characteristics of the TME, the ESTIMATE algorithm 37, a method inferring tumor purity and stromal and immune cell from tumor samples based on bulk transcriptomic profile, was performed to assess tumor purity, ESTIMATE score, immune score, and stromal score. Besides, the information of immunomodulators including MHC signatures, receptors, chemokines, and immune-stimulators was collected from the previous studies 38. To further deconstruct the immunological status of each patient, a set of signature genes of 29 immune cell types and immune-related pathways 39 was used to estimate the infiltration levels of different immune cell populations and the activities of immune-related pathways and functions of each patient were calculated by utilizing the single-sample gene sets enrichment analysis (ssGSEA) in the R package “GSVA” 40.

### Cell-cell communication analysis

Cell-cell communications mediated by ligand-receptor complexes were critical to diverse biological processes, such as inflammation and tumorigenesis. To investigate the molecular interaction networks between different cell types, we used “CellPhoneDB” 41, a software to infer cell-cell communication from the combined expression of multi-subunit ligand-receptor complexes, to analyze the interactions between tumor cells and microenvironment cell subpopulations. The ligand-receptor pairs with a P value < 0.05 were remained for the assessment of relationship among different cell clusters.

### Immunohistochemistry and semi-quantitative analysis

Lung cancer tumor microarray (TMA) HLugC120PT01, which was purchased from Outdo BioTech, contained 60 paired tumor and para-tumor samples. A total of 58 tumor samples were included in our research after removing the samples separated from the TMA, and the detailed clinic parameters of enrolled in-house patients were exhibited in Table S2. The use of the TMA was approved by the Clinical Research Ethics Committee in Outdo Biotech (Shanghai, China). The TMA was submitted for immunohistochemistry (IHC) assay to define the protein expression of CTNND1 in tumor and para-tumor tissues. The sections were then washed with xylene for three 5-min. The sections were rehydrated by successive washes in 100, 90 and 70% graded ethanol. Hydrogen peroxidase (0.3%) was used to block endogenous peroxidase activity for 20 min. The EDTA antigen repair solution was used for antigen repair. The primary antibody utilized in the study was anti-CTNND1 (1:200 dilution, Cat. sc-23873, Santa Cruz) and anti-CD8A (ready-to-use, Cat. PA577, Abcarta). Antibody staining was visualized with DAB and hematoxylin counterstain. Stained TMA was evaluated to define CTNND1 expression by two independent senior pathologists according to the immunoreactivity score standard 42. For the assessment of tumor-infiltrating CD8+ T cells, two senior pathologists estimated the CD8 score according to the criterion established by The Cancer Genome Atlas Network 43. A CD8 score defined as the sum of the distribution and density scores (0-6) was calculated for each case. Samples with the CD8 score ≥ 3 (3, 4, 5, 6) are considered to be immune-hot, and samples with the CD8 score ≤ 2 (0, 2) are considered to be immune-cold.

### Statistical analysis

All statistical analyses were handled using R software (version 4.0.4). The significant difference in continuous variables between the two groups was assessed using the Wilcoxon rank-sum test, while fisher exact test was used to measure the difference among categorical variables. For all analyses, a two-paired p-value < 0.05 was deemed to be statistically significant, and labeled with *p-value < 0.05, **p-value < 0.01, ***p-value < 0.001, and ****p-value < 0.0001.

## Results

### Anoikis-related genes expressed specifically in tumor cells of NSCLC at single-cell atlas

**Figure 2:**
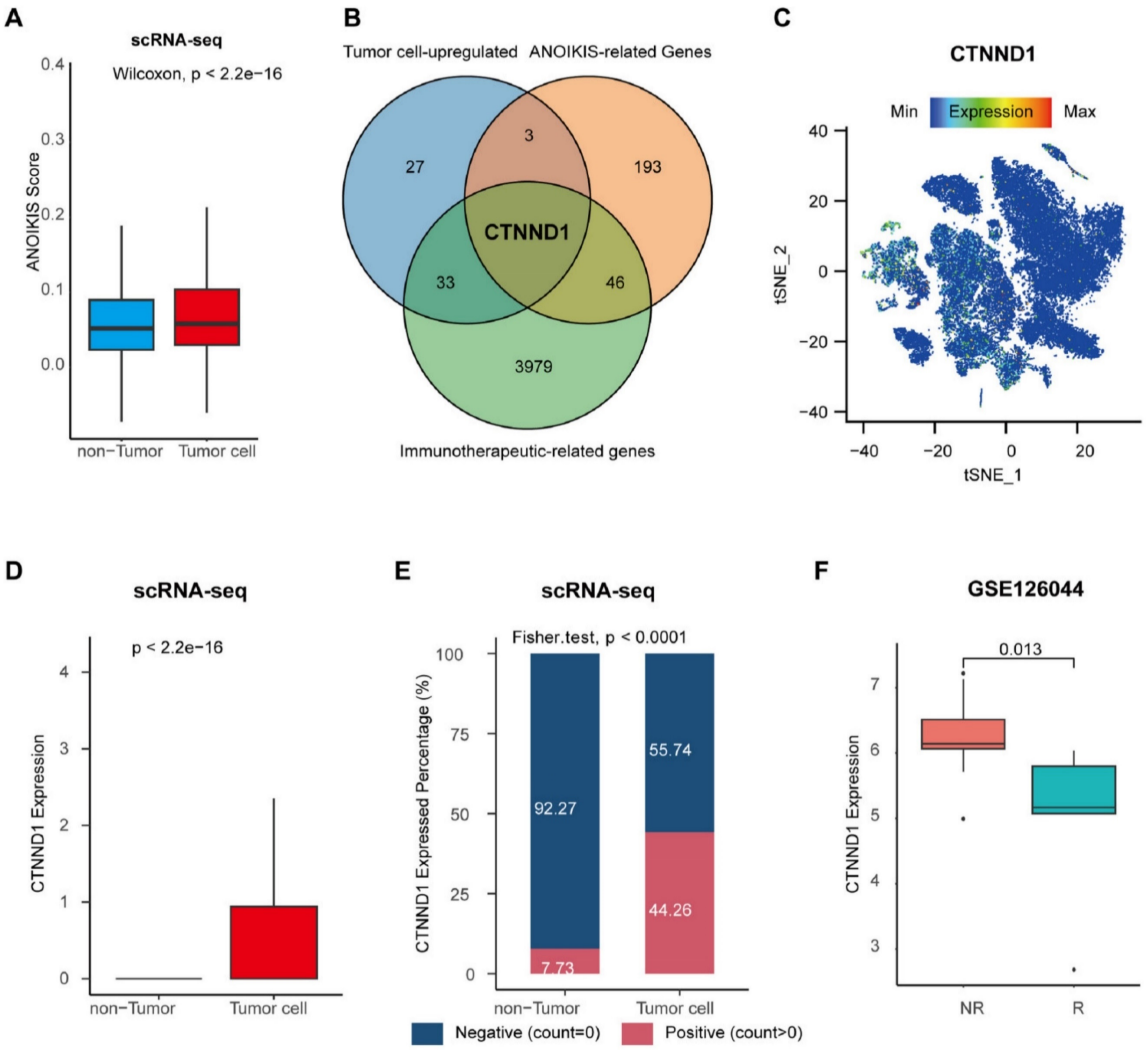
** Identification of the tumor cell-specific anoikis-related genes.** (A) Boxplot showing the anoikis scores between tumor and non-tumor cells. (B) Venn diagram of overlapping genes in tumor cell-upregulated genes identified in the scRNA-seq datasets, anoikis-related genes from the Harmonizome database, and immunotherapeutic-related genes recognized in the GSE126044 cohort. (C) Expression levels of CTNND1 overlaid on the t-SNE representation. (D) Comparing the expression levels of CTNND1 between tumor and non-tumor cells. (E) Stacking bar chart showing the fraction of cells expressed or not expressed CTNND1. (F) Comparing the expression levels of CTNND1 between NSCLC patients who received (R) or not received remission after immunotherapy (NR) in the GSE126044 cohort. Wilcoxon rank-sum test was performed to measure the difference between the two groups. The data in Figure 2A-E were downloaded from the GSE150660, the GSE127465, and the GSE117570 datasets. The data in Figure 2F were downloaded from the GSE126044 dataset.

Using the “Seurat” package to preprocess the single cell transcriptome data, a total of 47,359 cells were obtained (Figure 1A-1B). Markers of various cell types of NSCLC were collected, and the cells were annotated. The cells were divided into epithelial cells (EPCAM), T cells (CD3E), B cells (CD19), fibroblasts (COL1A1), endothelial cells (VWF), myeloid cells (CD14), and plasma cell (SLAMF7) (Figure 1C). Perform feature recognition on each annotated cluster of cell subpopulations to verify the accuracy of our cell type annotation (Figure 1D-1E). Furthermore, we used the ssGSEA algorithm to calculate the ANOIKIS score for the genes related to anoikis, and compared their differences between tumor cells and non-tumor cells identified previously. The results showed that the ANOIKIS score was significantly higher in tumor cells than in non-tumor cells (Figure 2A), thus confirming that anoikis is an important feature of tumor cells of NSCLC.

### CTNND1 was identified as a tumor specific biomarker of immunotherapy for NSCLC

In order to explore the characteristic markers, and remodeling effect of tumor anoikis on the immune microenvironment of NSCLC, we analyzed the immune cohort data GSE126044. We obtained 3979 differential genes significantly related to the efficacy of immunotherapy through differential analysis. Next, we intersected immunotherapy-associated differential genes with anoikis-related genes, and the genes significantly expressed in tumor cells, obtaining a special biomarker: CTNND1, which can be inferred as an immunotherapy and tumor anoikis related biomarker (Figure 2B). The CTNND1 gene was validated in single cell atlas, and the results showed that its expression in tumor cells was significantly higher than that in non-tumor cells (Figure 2C-D). We binarized tumor cells based on whether the expression of CTNND1 was greater than 0, and compared the proportion of positive and negative cell rates in tumor and non-tumor cells. The results showed that the rate of CTNND1+ cells was significantly higher in the tumor cells than in non-tumor cells (Figure 2E). Finally, in the GSE126044 data, the expression of CTNND1 in the immunotherapy sensitive group was significantly higher than that in the ineffective group (Figure 2F).

**Figure 3:**
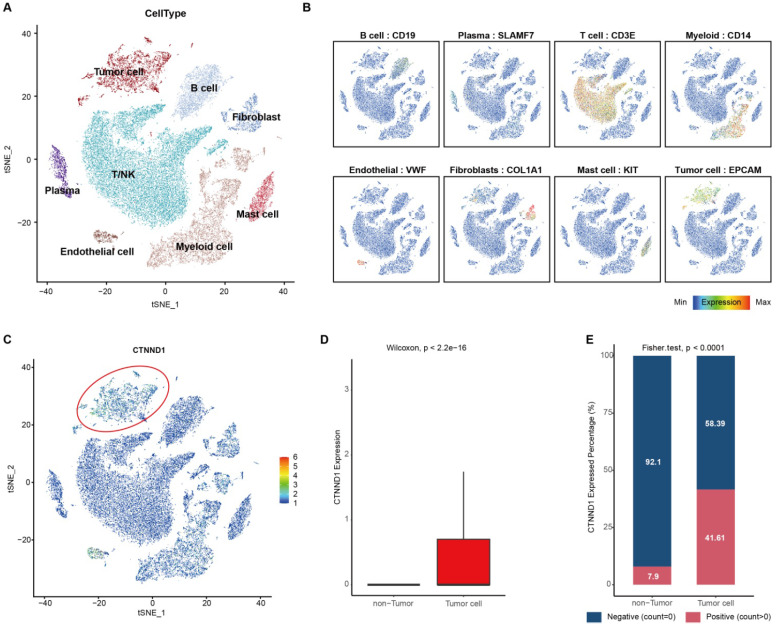
** Integrated scRNA-seq analysis of tumor tissues from NSCLC patients in the GSE131907 datasets.** (A) t-SNE visualization of cell types annotated by classical gene markers. (B) Expression levels of known markers for specific cell types overlaid on the t-SNE representation. (C) Expression levels of CTNND1 overlaid on the t-SNE representation. (D) Comparing the expression levels of CTNND1 between tumor and non-tumor cells. (E) Stacking bar chart showing the fraction of cells expressed or not expressed CTNND1. The data in Figure 3 were downloaded from the GSE131907 dataset.

Furthermore, another scRNA-seq dataset (GSE131907) including 11 NSCLC patients was used to validate the results found at the single-cell levels. Unsupervised clustering and cell annotation, we classified the cells into eight cell types (Figure 3A-3B, Supplementary Figure 1A-1B). Consistently, CTNND1 almost expressed on tumor cells (Figure 3C-3D). Meanwhile, after binarizing the cells into CTNND1+ and CTNND1- groups, the rate of CTNND1+ cells were significantly higher in the tumor cells than in non-tumor cells (Figure 3E), further supporting the viewpoint that CTNND1 was the biomarker of tumor cells.

### CTNND1+ tumor cells communicated with microenvironment subpopulations frequently

**Figure 4:**
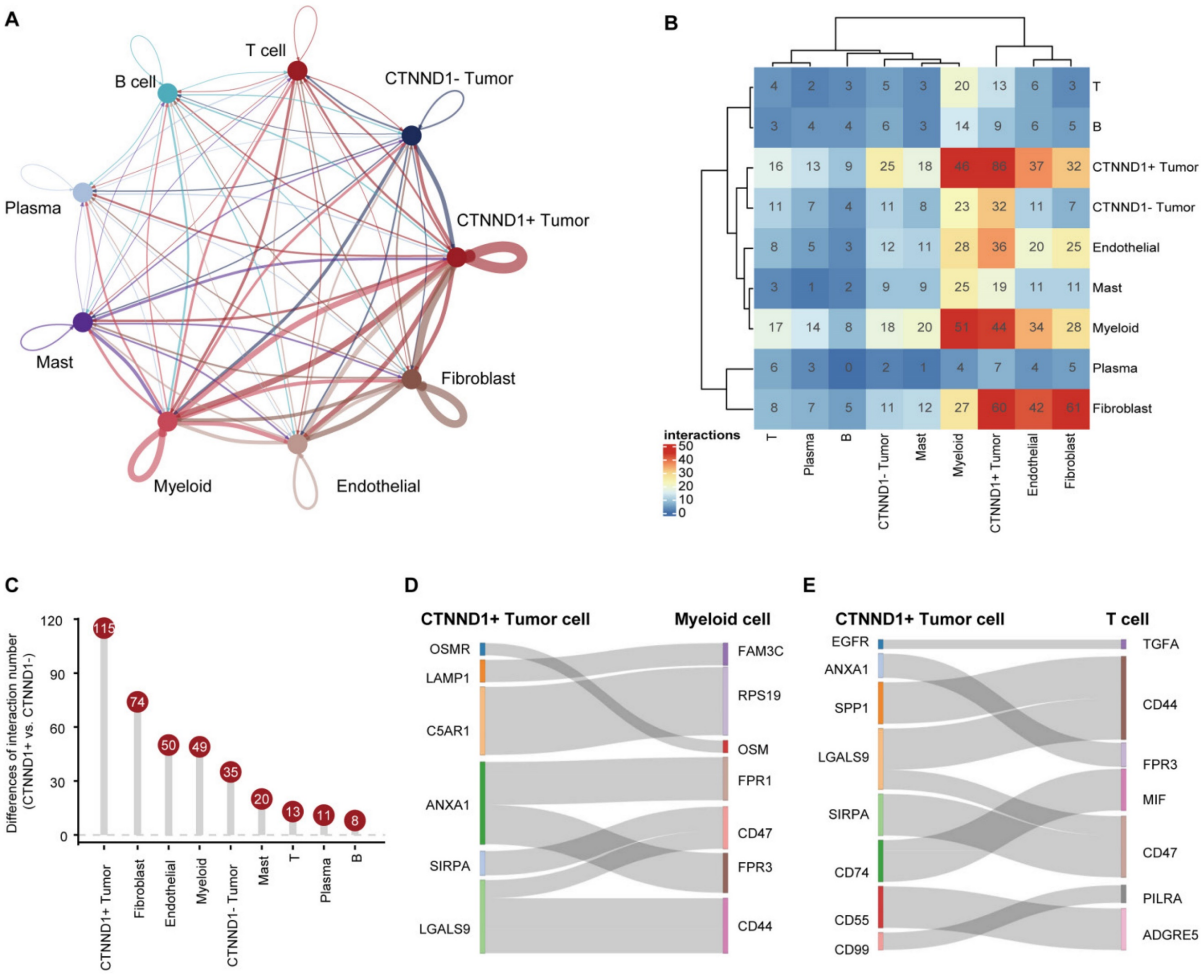
** Cell-cell communications between CTNND1+/- tumor cells and microenvironment subpopulations.** (A) The interaction number of CTNND1+/- tumor cells and microenvironment subpopulations. The thickness of the line represents the interaction number between the subpopulations estimated by CellPhoneDB. (B) Heatmap showing the interactions among these cell types. (C) The difference of the number of ligand-receptor interactions between CTNND1+ and CTNND1- tumor cells. (D) The inhibitory interactions between CTNND1+ tumor cells and myeloid cells. (E) The inhibitory interactions between CTNND1+ tumor cells and T cells. The data in Figure 4 were downloaded from the GSE150660, GSE127465, and GSE117570 dataset.

Having observed that the expression of CTNND1 in the immunotherapy sensitive group was significantly lower than that in the ineffective group, we next sought to investigate the correlations between CTNND1 expression and the TME status of the NSCLC patients. Benefiting from the advantages of scRNA-seq technology, we could perform a high-resolution dissection of interactions among various subgroups of CTNND1+/- tumor cells and microenvironment subpopulation in the NSCLC patients based on the combining expression of multi-subunit ligand-receptor complexes. The number of interactions among different subpopulations was compared between CTNND1+ and CTNND1- tumor cells. Results showed that the CTNND1+ tumor cells presented significantly more interactions than CTNND1- tumor cells (Figure 4A-4B). Notably, we calculated the difference in interaction numbers among various CTNND1+/- tumor cells and microenvironment subpopulations. The CTNND1+ tumor cells showed more frequently crosstalk with other subpopulations, especially the immune subsets (Figure 4C). Results from the GSE131907 scRNA-seq dataset was in-kept with these findings (Supplementary Figure 2). These results collectively suggested that compared with CTNND1- tumor cells, CTNND1+ tumor cells had activated cell-cell communications, especially interactions with immune cells, which potentially take part in the formation of an immunosuppressive TME 44.

We further identified the significant ligand-receptor interactions between CTNND1+ tumor cells and immune subsets using the “CellphoneDB” tool 41 (Figure 4D-4E). Results showed that CTNND1+ tumor cells communicated with myeloid cells via ANXA1-FPR1/3, SIRPA-CD47, and LGALS9-CD44, which have been reported to involve in the inhibition of anti-tumor response 45-47. In addition, the CTNND+ tumor cells mediated the dysfunction of T cells via inhibitory ligand-receptor pairs, such as ANXA1-FPR3 48, 49. In addition, compared with CTNND1- tumor cells, CTNND1+ tumor cells showed the highest cell-cell communication strength with stromal cells (fibroblasts and endothelial cells) (Figure 4B-4C), and presented significantly more specific interactions with stromal cells (Supplementary Figure 2A-2B). For example, CTNND1+ tumor cells communicated with stromal cells via many ligand-receptor pairs, such as COL1A1_a1b1 complex, which involved in the activation and differentiation of endothelial and fibroblasts 50, 51, suggesting that the accumulation of CTNND1+ tumor cells will be accompanied by the enrichment of stromal cells, leading to the formation of desert TME. Besides, our results also showed that some interactions associated with tumor stemness, chemoresistance, and angiogenesis, such as WNT7B-FZD4 and NOTHC1-JAG1 52, 53, were detected between CTNND1+ tumor cells and stromal cells (Supplementary Figure 3A-3B). Some studies proved that selectively blocking JAG/NOTCH can disrupt angiogenesis by unique mechanisms to inhibit tumor growth 54, 55. Totally, compared with CTNND1- tumor cells, CTNND1+ tumor cells interacted with microenvironment subpopulations frequently, suggesting that the complex crosstalk between CTNND1+ tumor cells and other subpopulations will contribute to the formation of TME.

### High expression of CTNND1 was related to immunosuppressive status of NSCLC

**Figure 5:**
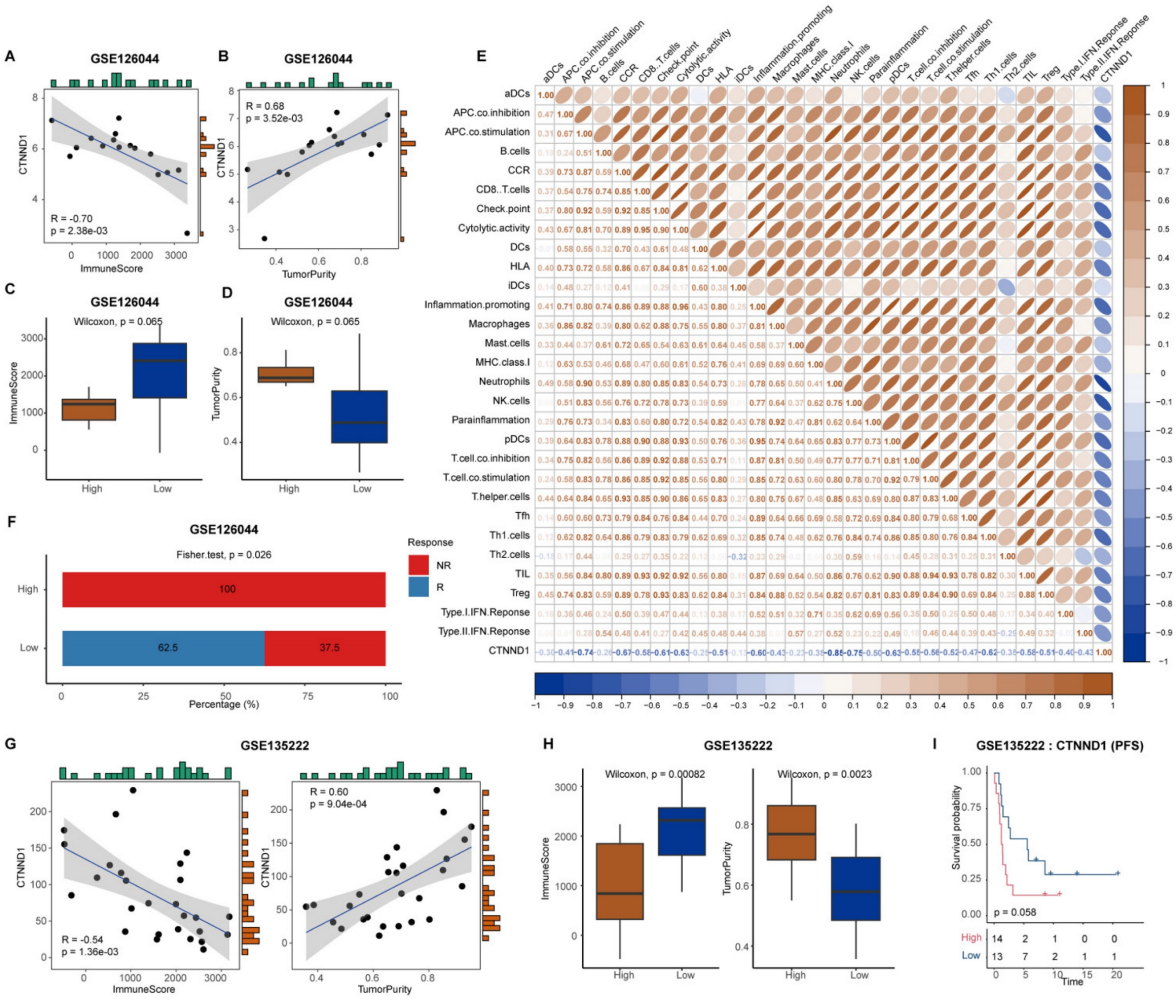
** Correlation between CTNND1 expression and immunological characteristics.** (A-B) Correlation between CTNND1 expression and ImmuneScore (A) and tumor purity (B) in the GSE126044 cohort. (C-D) Comparing the ImmuneScore (C) and tumor purity (D) between CTNND1-high and low groups in the GSE126044 cohort. (E) Correlation between CTNND1 expression and levels of immunological characteristics in the GSE126044 cohort. (F) Stacking bar chart showing the fraction of R and NR patients in the CTNND1-high and low groups. (G) Correlation between CTNND1 expression and ImmuneScore (left) and tumor purity (right) in the GSE135222 cohort. (H) Comparing the ImmuneScore (left) and tumor purity (right) between CTNND1-high and low groups in the GSE135222 cohort. (I) Kaplan-Meier analysis in term of progression-free survival (PFS) in the GSE135222 cohort. Patients were divided into two groups based on the median expression of CTNND1. Wilcoxon rank-sum test was performed to measure the difference between the two groups.

Next, we explored the effect of CTNND1 on the remodeling of immune microenvironment of NSCLC. As shown in Supplementary Figure 4A-4D, we found that CTNND1 expression showed no significant differences between patients with different clinic parameters, suggesting that clinical parameters did not influence the expression levels of CTNND1. Then, we calculated the tumor purity and immune infiltration score in each sample through ESTIMATE, and carried out correlation analysis with the expression of CTNND1. The results showed that the expression of CTNND1 was significantly positively correlated with the tumor purity, and significantly negatively with tumor purity (Figure 5A-B). The samples were divided into high and low expression groups based on the median expression of CTNND1, and the results showed that the immune infiltration score of the high expression patients was significantly lower than that of the low expression patients (Figure 5C-D). Furthermore, we investigated the relationship between CTNND1 and the degree of infiltration of various types of immune cells, which showed that the infiltration degree of immune cells was negatively correlated with CTNND1 expression (Figure 5E). Meanwhile, patients with high expression of CTNND1 showed resistance to immunotherapy, while 62.5% of patients with low expression showed sensitivity (Figure 5F). The results of the GSE135222 dataset also confirmed this conclusion (Figure 5G-H), and survival analysis showed that patients with high CTNND1 expression had poorer progression-free survival compared to those with low expression (Figure 5I). In addition, in other epithelial cell carcinomas, such as gastric carcinoma, breast cancer and melanoma, patients in the CTNND1-high group also showed poor immunotherapeutic response than those in the CTNND1-low group (Supplementary Figure 5A-5C).

**Figure 6:**
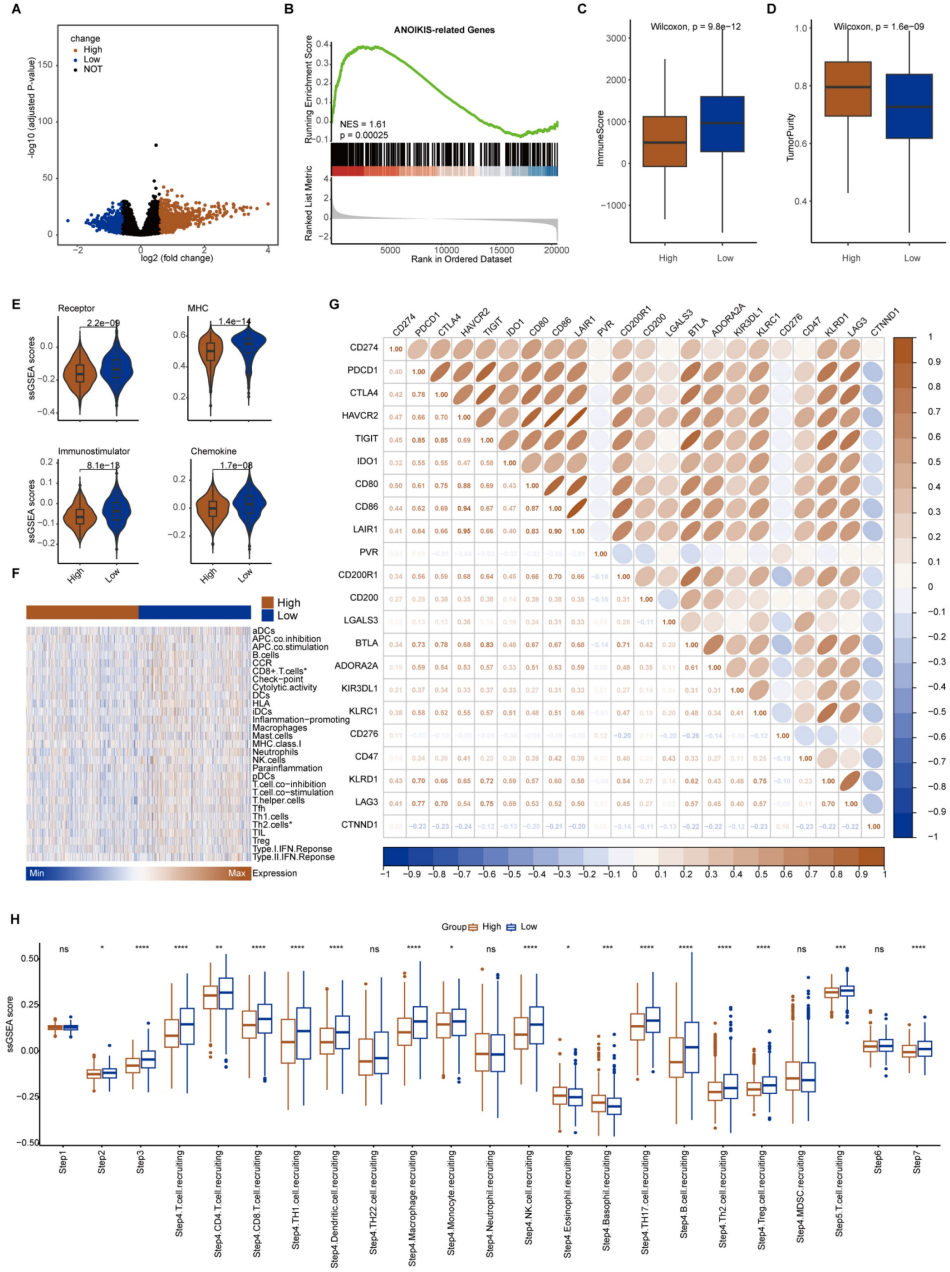
** Immunological characteristics between CTNND1-high and CTNND1-low groups in the TCGA cohort.** (A) Volcano plot showing the up-regulated (brown) and down-regulated (blue) genes of the CTNND1-high group in the TCGA-NSCLC cohort. (B) A representative gene set enrichment analysis plot showing significant upregulated ANOIKIS-related genes in the CTNND1-high group versus the CTNND1-low group in the TCGA cohort. (C-D) Comparing the ImmuneScore (C) and tumor purity (D) between CTNND1-high and low groups in the TCGA cohort. (E) Comparison of the enrichment scores of receptors, MHC, immunostimulator, and chemokine between CTNND1-high and CTNND1-low groups. (F) Heatmap showing the enrichment scores of immune subpopulations and immune-related signaling pathways. (G) The correlation between CTNND1 expression and some conventional immune checkpoint inhibitors in the TCGA cohort. (H) Comparing the enrichment scores of each step in the cancer immunity cycle between CTNND1-high and low groups. Wilcoxon rank-sum test was performed to measure the difference between ang two groups.

**Figure 7:**
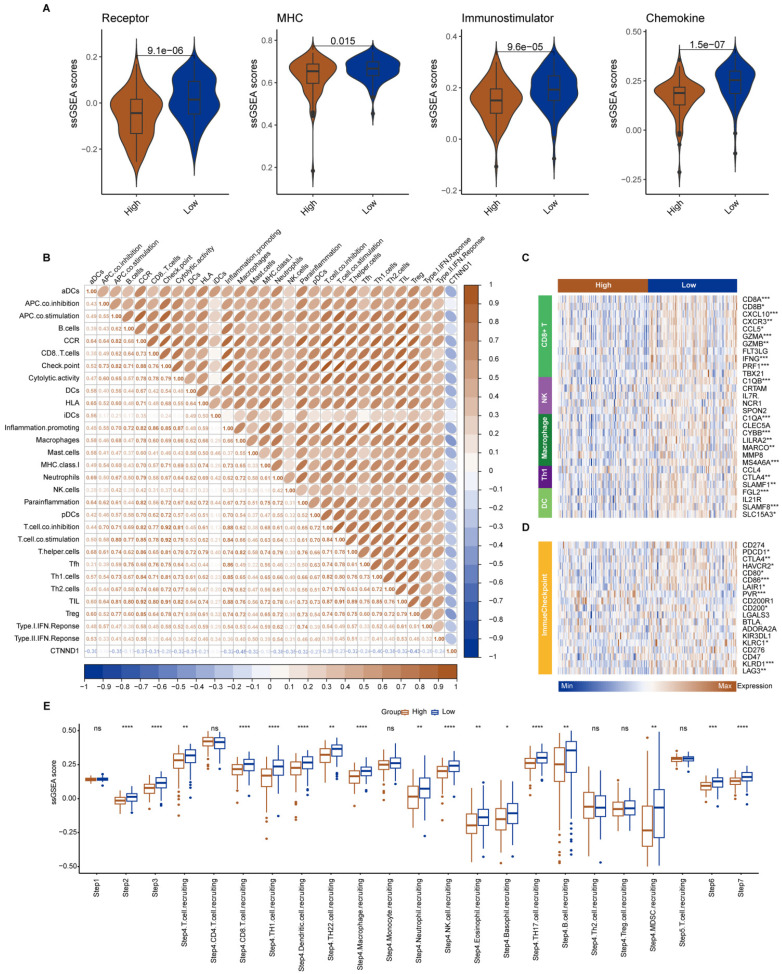
** Immunological characteristics between CTNND1-high and CTNND1-low groups in the GSE42127 cohort.** (A) Comparison of the enrichment scores of receptors, MHC, immunostimulator, and chemokine between CTNND1-high and CTNND1-low groups. (B) The correlation between CTNND1 expression and the enrichment scores of immune subpopulations and immune-related signaling pathways. (C-D) Heatmap showing the expression levels of conventional markers of immune cells (C) and immune checkpoint inhibitors (D). (E) Comparing the enrichment scores of each step in the cancer immunity cycle between CTNND1-high and low groups. Wilcoxon rank-sum test was performed to measure the difference between the two groups.

The median expression value of CTNND1 in NSCLC cases in TCGA was used as the threshold to divide the samples into two groups: CTNND1 high and CTNND1 low, and then performed differential analysis. The volcano plot shows up-regulated and down-regulated genes. The results of functional enrichment analysis showed that the ANOIKIS-related pathway in the CTNND1 high groups were significantly upregulated (Figure 6A-B). The samples from TCGA also showed the feature of low immune infiltration in the CTNND1 high expression group (Figure 6C). The levels of immune receptor activation, antigen presentation, immune activation, and cytokine between high- and low-CTNND1 expression groups were then compared by ssGSEA scores, in order to further investigate the relationship between CTNND1 and immune function. The results showed that the immune function of the CTNND1 high group was significantly lower than patients of the low expression (Figure 6E). Comparing the activation levels of various types of immune cells between the two groups, we found that the abundance of all immune cells was lower in high CTNND1 group, especially CD8+T cells (Figure 6F, Supplementary Figure 6 and Figure 7). To further investigate the role of this marker in immune suppression, we investigated the relationship between CTNND1 and various immune checkpoints, and the results showed a negative correlation with most immune checkpoints, indicating that high expression patients were ineffective in immunotherapy (Figure 6G). Finally, we compared the enrichment scores of various steps in the cancer immune cycle between CTNND1 high and low groups, our finding suggested a significant negative correlation between CTNND1 expression and various processes of the immune cycle (Figure 4I). To validate our conclusion, we conducted further analysis on the validation set GSE42127 (Figure 7). Our results indicate that CTNND1 expression is significantly negatively correlated with immune cells abundance and immune-related signaling pathways. Additionally, we observed that the expression of immune checkpoints in the high expression group is significantly lower than that in the low expression group, which may indicate ineffective immunotherapy.

Last but not least, given of the importance of genomic variants in diagnosis and therapeutic guidance, we compared the mutations between the CTNND1-high and low groups (Table S3). Results showed that some genomic variants, such as TP53 56 and KMT2D 57, associated with worse clinical outcomes and tumor invasion were enriched in CTNND1-high group, while CTNND1-low groups had higher mutant frequency of some gene associated with immune infiltration and favorable outcomes, such as CACNA1C 58 and LMO7 59 (Supplementary Figure 8). These results were consistent with the phenotypes found at the transcriptional levels, and further indicated that for those patients in the CTNND1-high group, targeting some mutations, such as TP53 can benefit patients.

### Validation of CTNND1 expression and immuno-correlation in NSCLC

**Figure 8:**
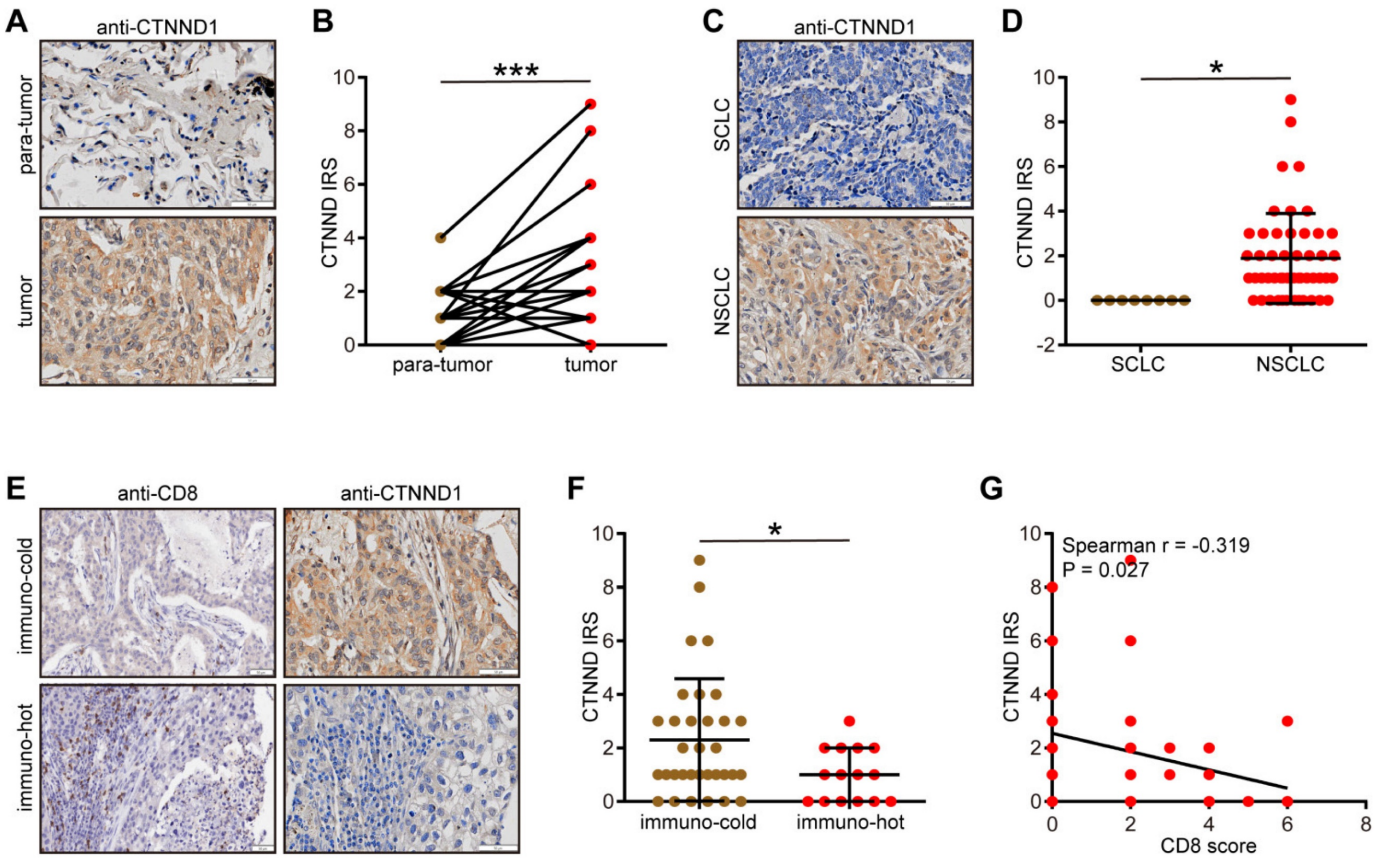
** Validation of CTNND1 expression and its correlations with the TME features in the in-house cohort.** (A) Representative images uncovering the expression patterns in para-tumor and tumor tissues. (B) Semi-quantitative analysis for (A). (C) Representative images uncovering the expression patterns in NSCLC and SCLC tumor tissues. (D) Semi-quantitative analysis for (C). (E) Representative images uncovering the expression patterns in immuno-cold and immuno-hot tumor tissues. (F) Semi-quantitative analysis for (E). (G) Correlation between CTNND1 expression and CD8 score.

We next validated the expression patterns of CTNND1 in the in-house cohort. We found that CTNND1 was highly expressed in tumor tissues compared with para-tumor tissues (Figure 8A-8B) and CTNND1 was specifically expressed in NSCLC (Figure 8C-8D). We also divided NSCLC as immuno-hot and immune cold tumors based on the CD8 score. Obviously, CTNND1 was decreased in immuno-hot tumors (Figure 8E-8F) and negatively correlated with the CD8 score (Figure 8G). Overall, the negative correlation between CTNND1 expression and T cell infiltration could be validated in the in-house cohort, which largely increases the credibility of results by public cohorts.

## Discussion

NSCLC is a common malignant tumor with a high incidence rate, and predilection to metastasize. As we all know, blocking immunosuppressive checkpoint inhibitors such as CTLA-4, programmed cell death protein-1 (PD-1) and related programmed death ligand 1 (PD-L1) has revolutionized the first-line treatment of advanced NSCLC. Both the Food and Drug Administration (FDA) and the National Medical Products Administration (NMPA) of China have approved multiple indications for ICI in clinical practice of NSCLC, including PD-1 inhibitors, PD-L1 inhibitors, and CTLA-4 inhibitors. Most of them are immunotherapy combinations 60-63. Although this new treatment method has brought survival benefits to some patients, there are still some patients who cannot achieve effective relief. Thus, biomarkers that could distinguish patients benefitting from immunotherapy are critical to the precise application of immunotherapy 64-66.

Apoptosis of tumor cells can lead to drug resistance, thereby mediating the survival of tumor cells during circulation in the bloodstream, which is crucial for the progression of metastasis. Jin et al. found that the PLAG1 GDH1 axis promotes resistance to apoptosis and tumor metastasis in LKB1 deficient cancers through the CamKK2 AMPK signaling pathway 67. Meanwhile, studies have shown that CPT1A mediated fatty acid oxidation can promote metastasis of colorectal cancer cells by inhibiting nest loss apoptosis 68. In the gastric cancer cohort, Ye et al. found that nuclear MYH9 induced CTNNB1 transcription promotes anti nesting apoptosis and metastasis in gastric cancer cells 69. Anoikis has done a lot of research on tumor proliferation and metastasis 70-72, but its role in remodeling the TME and thus mediating tumor progression has not been in-depth studied. We focus on the role of anoikis play in tumor microenvironment and explore whether anoikis could mediate the regulation of immunization, thus affecting tumor proliferation and metastasis.

We distinguish malignant tumor cells from TME cells, including immune cells and stromal cells through single cell transcriptome data and cell annotation, and obtain specific markers of malignant tumor cells through differential expression analysis. Through the NSCLC immunotherapy queue, we screened biomarkers related to the efficacy of immunotherapy. The two gene sets were intersected with anoikis related genes to obtain CTNND1, which was identified as an immune regulation related anoikis tumor marker. We suspect that CTNND1 could reshape the TME by regulating anoikis, thus affecting the efficacy of immunotherapy. CTNND1 has been proved by many studies to be closely related to tumor proliferation and metastasis, such as colorectal cancer 73-75, breast cancer 76, 77, gastric cancer 78, liver cancer 79, etc. In addition, CTNND1 drives butyrophilin-like molecule loss and γδ T-cell exclusion, indicating the immuno-suppressive role of CTNND1 In colon cancer 80. However, there is no research on the regulation of this gene on TME in NSCLC at present, which could be a research hotspot in the future.

In order to explore its regulation on immune cells, we use a variety of algorithms to evaluate the immune infiltration of each sample, the activation of various types of immune cells, and the biological processes of immune action. Subsequently, correlation analysis was conducted between CTNND1 and them, and the results showed that the expression level of CTNND1 was significantly negatively correlated with the degree of immune infiltration, and all immune cells were inhibited by CTNND1. The above results suggested that CTNND1 has a comprehensive impact on immunosuppression, which runs through every stage of the immune response. Furthermore, patients with high expression of CTNND1 showed ineffective immunotherapy and poor progression free survival. Immune cell deficiency has been recognized as an important reason for ineffective immunotherapy 81, 82. Therefore, patients with high expression of CTNND1 performed the scarcity of immune cells may be the reason for ineffective immunotherapy. There is now evidence that immunotherapy can benefit NSCLC patients; However, due to the lack of full understanding of the Tumor microenvironment and immune cell infiltration in NSCLC, patients received immunotherapy without obtaining effective results. Thus, we conducted a comprehensive study on anoikis-related genes and delved into the prognosis and the TME characteristics of NSCLC. The study found that CTNND1 can be considered as a regulating upstream gene, targeting which is expected to activate the patient's immune system coincident with tumor cell killing.

## Conclusion

In this study, we investigated the expression of anoikis-related genes in NSCLC and identified genes specifically expressed in tumor cells. And we found that the expression of CTNND1 can guide immunotherapy, as a powerful prognostic marker for NSCLC. Finally, we explored the relationship between the expression of CTNND1 and the tumor immune microenvironment as well as immune regulatory biological processes. We found that the expression pattern of anoikis-related genes is closely related to the invasiveness of NSCLC, especially the CTNND1 gene, the expression of which can help to develop more personalized and precise treatment strategies in clinical practice.

## Supplementary Material

Supplementary figures.

Supplementary tables.
