# Supplementary material for: Anoikis-related CTNND1 is associated with immuno-suppressive tumor microenvironment and predicts unfavorable immunotherapeutic outcome in non-small cell lung cancer
Source: J Cancer. 2024 Jan 1;15(2):317–31. doi: 10.7150/jca.89542 (PMC10758022; doi:10.7150/jca.89542)
Supplement: Supplementary file 1 — Supplementary figures. [file jcav15p0317s1.pdf]

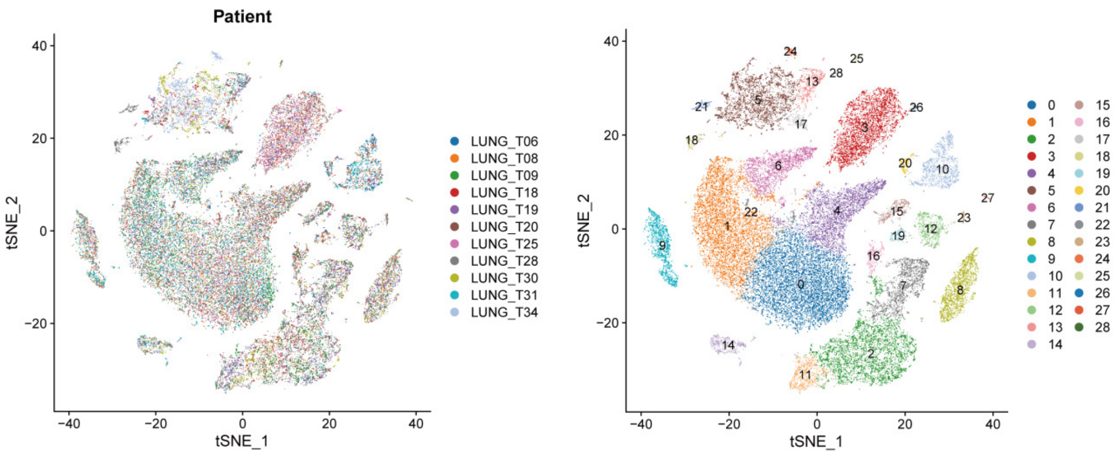

Supplementary Figure 1. Unsupervised clustering of the 11 NSCLC patients from the GSE131907 dataset.

(A) t-SNE visualization of single cells passed quality controls, colored by 11 NSCLC patients. (B) The unsupervised clustering of cells in the GSE131907 dataset.

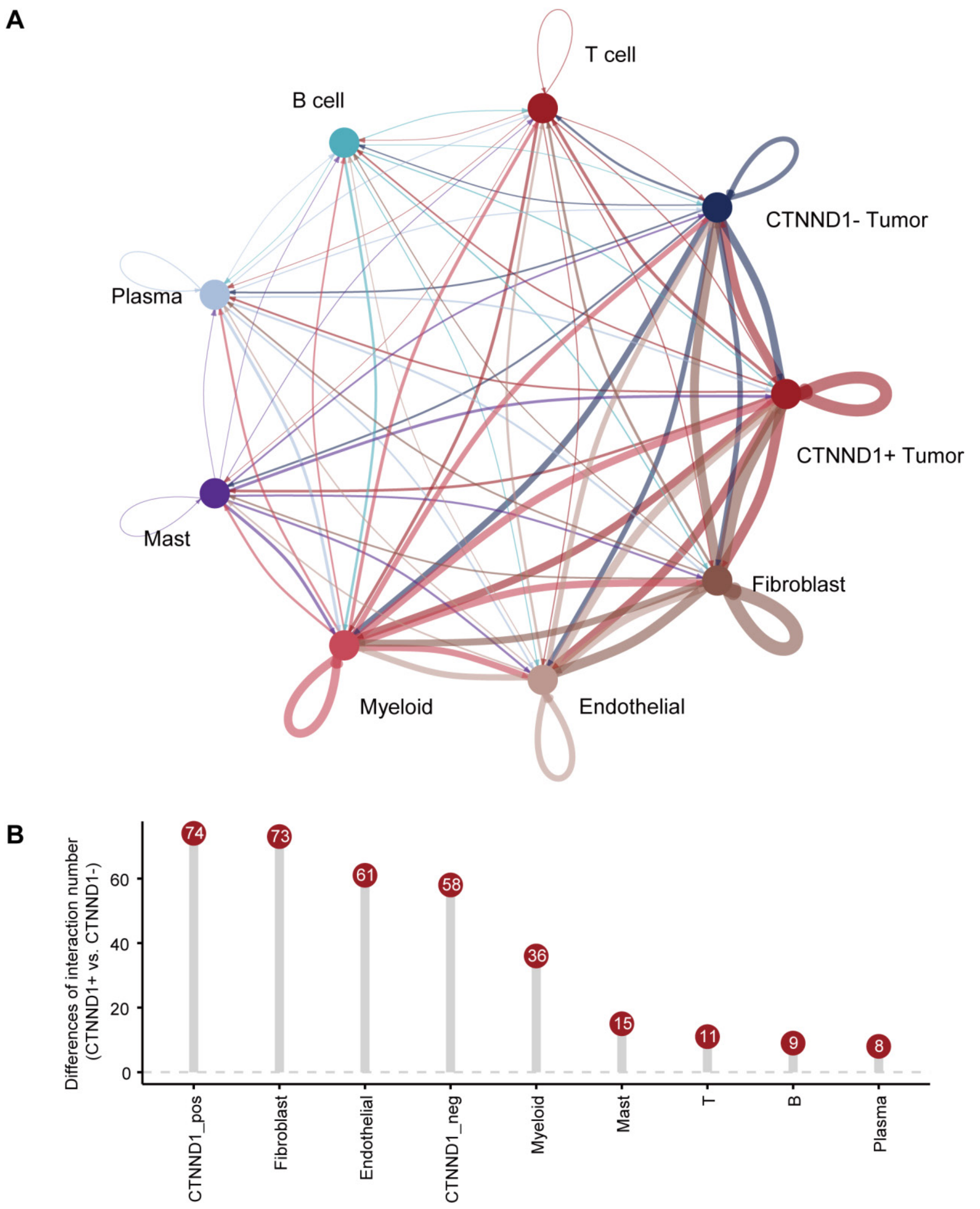

Supplementary Figure 2. Cell-cell communications between CTNND1+/- tumor cells and microenvironment subpopulations in the GSE131907 dataset.

(A) The interaction number of CTNND1+/- tumor cells and microenvironment subpopulations. The thickness of the line represents the interaction number between the subpopulations estimated by CellPhoneDB. (B) The difference of the number of ligand-receptor interactions between CTNND1+ and CTNND1- tumor cells.

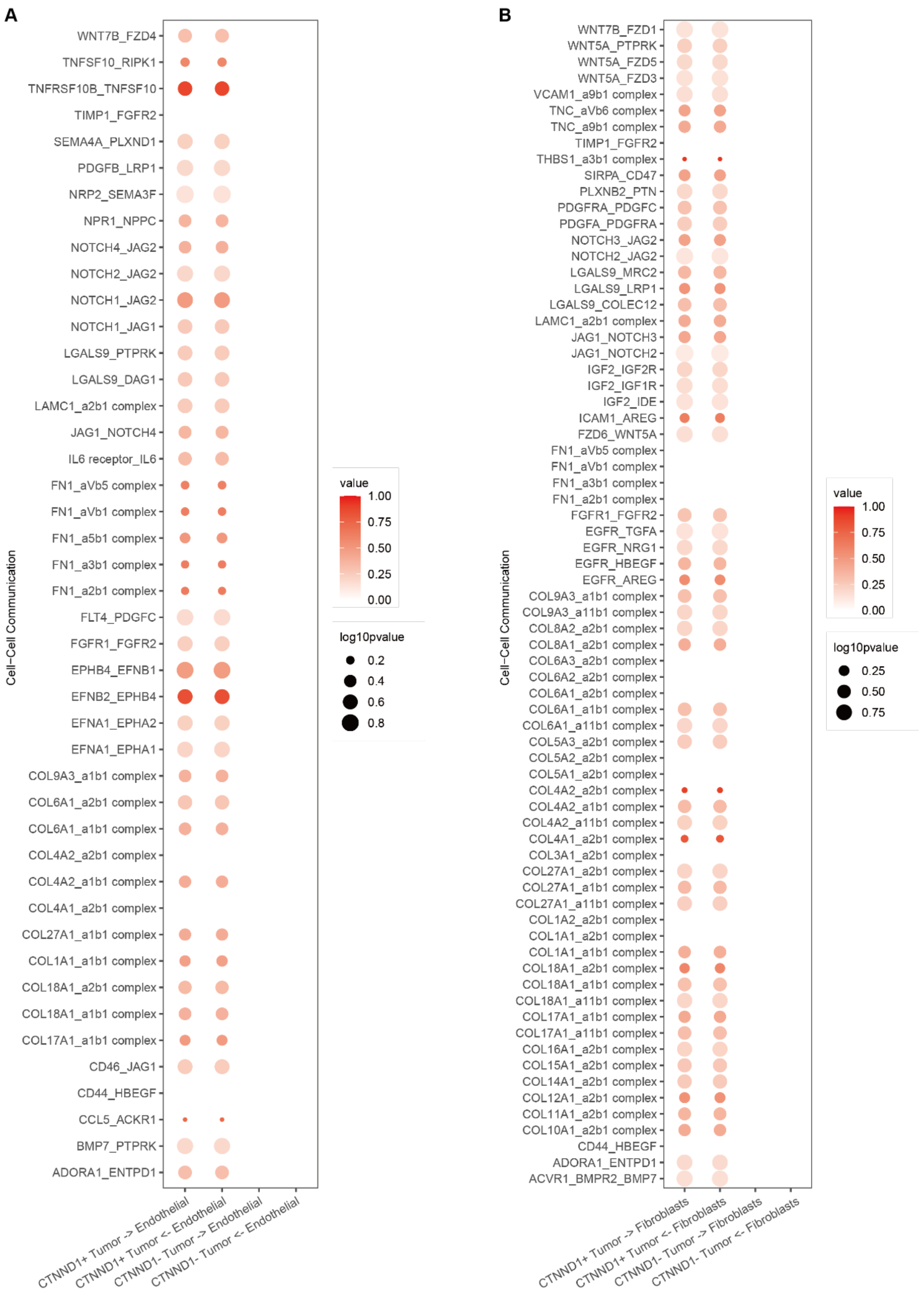

Supplementary Figure 3. Cell-cell communications between tumor and stromal cells. (A) The specific ligand-receptor interactions between tumor and endothelial cells. (B) The specific ligand-receptor interactions between tumor and fibroblasts.

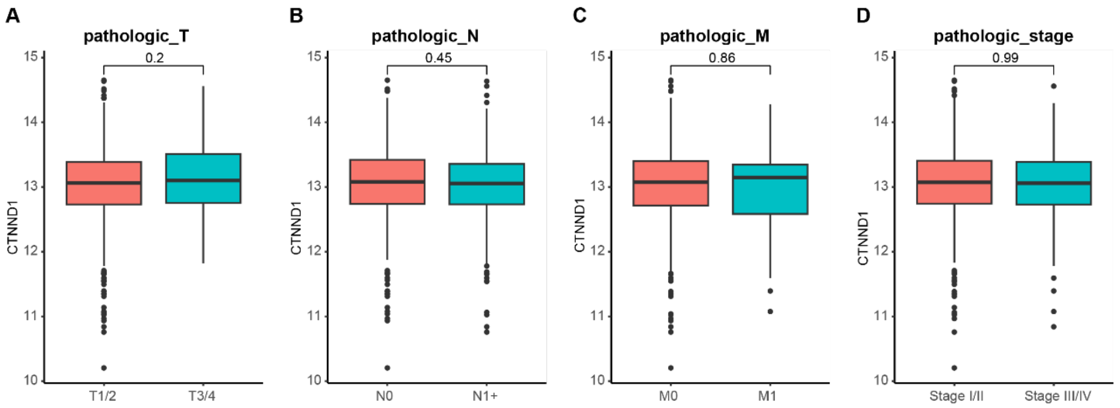

Supplementary Figure 4. Comparison of CTNND1 expression between patients with different clinic parameters. (A-D) Comparison of CTNND1 expression between patients with different T stage (A), N stage (B), M stage (C) clinical stages (D).

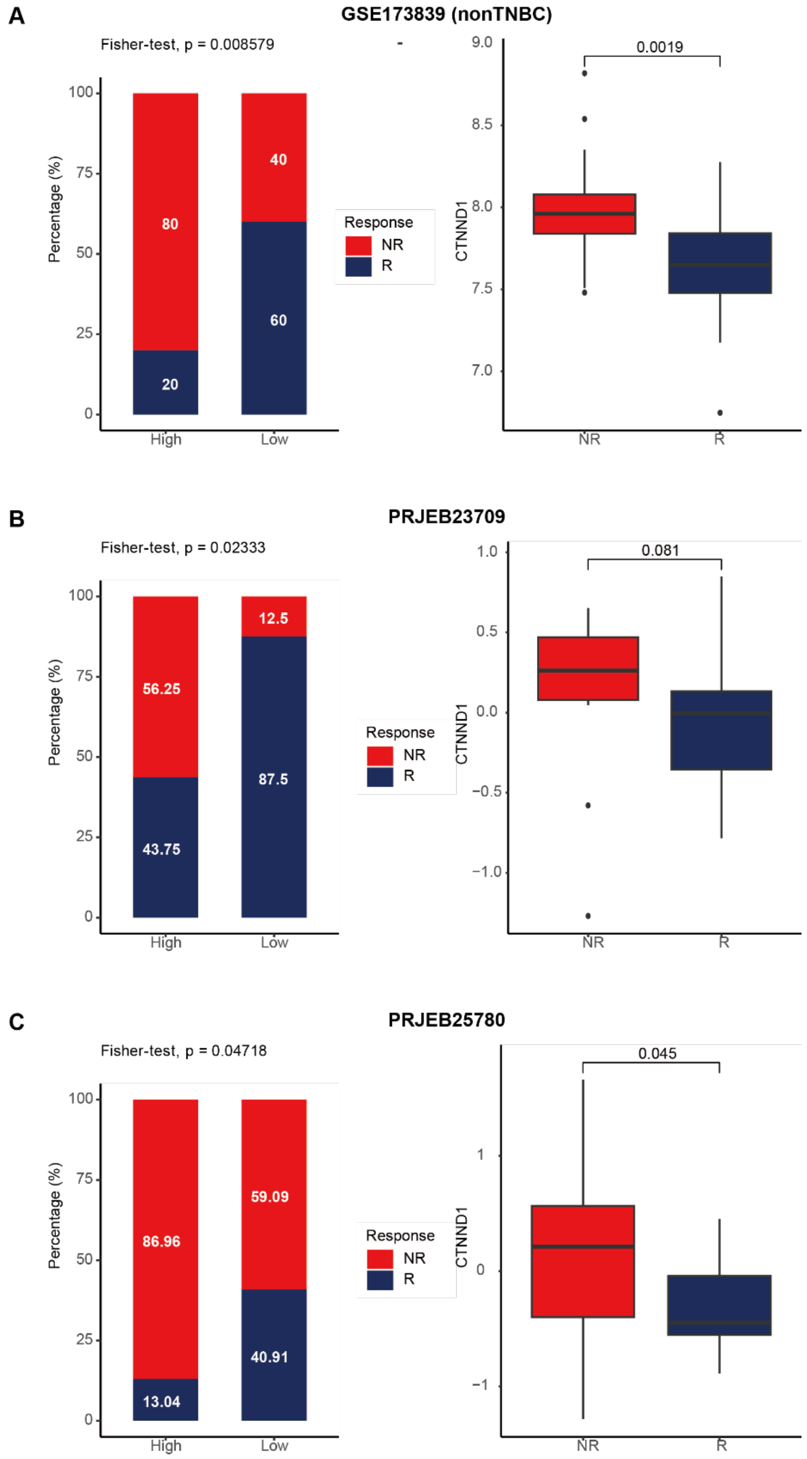

Supplementary Figure 5. Comparison of CTNND1 expression between patients with different therapeutic response. Left: Stacking bar chart showing the fraction of responders and non-responders in the CTNND1-high and low groups. Right: Comparing CTNND1 mRNA between responders and non-responders. R: responders, NR: non-responders.

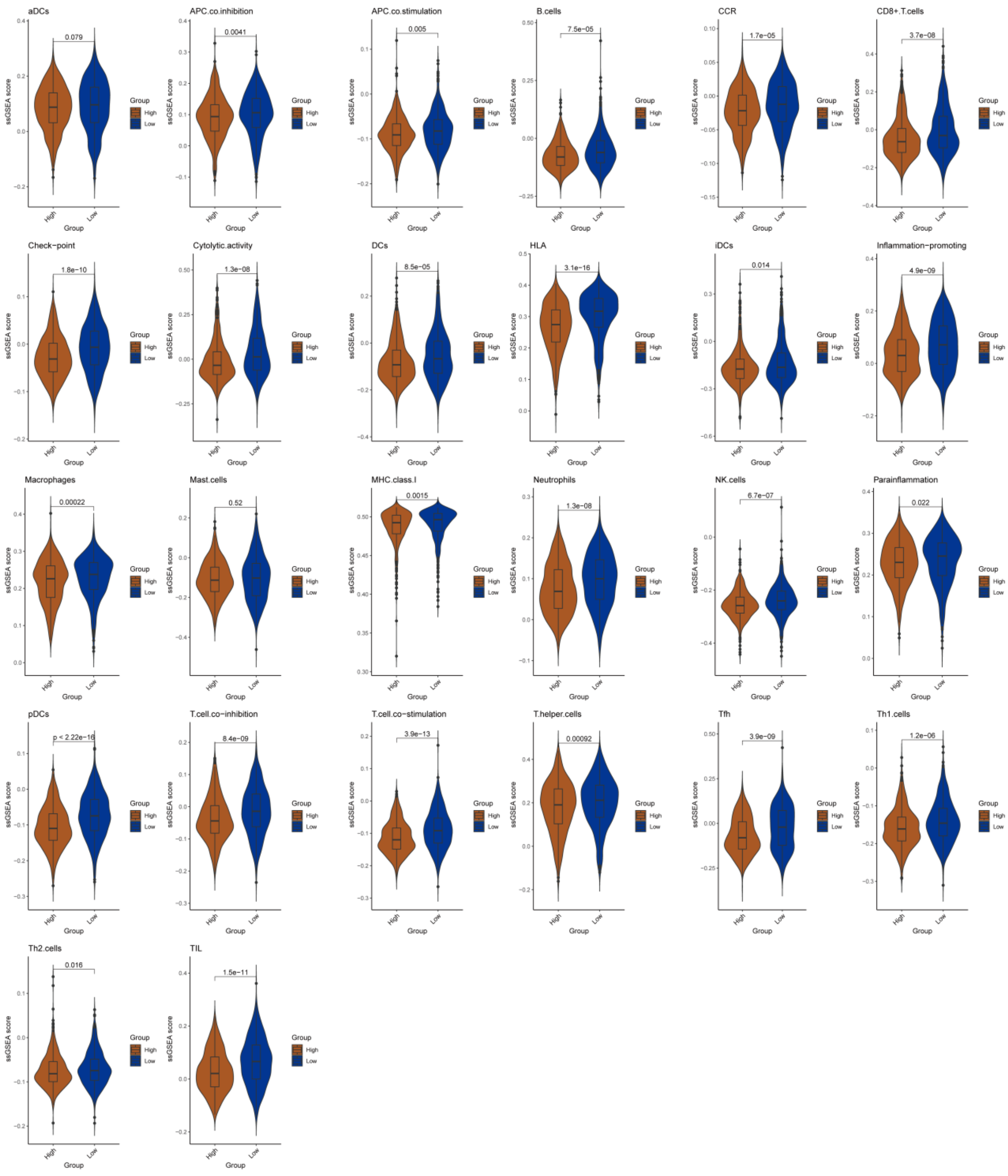

Supplementary Figure 6. Comparison of the 29 immunological characteristics between the CTNND1-high and low groups in the TCGA cohort.

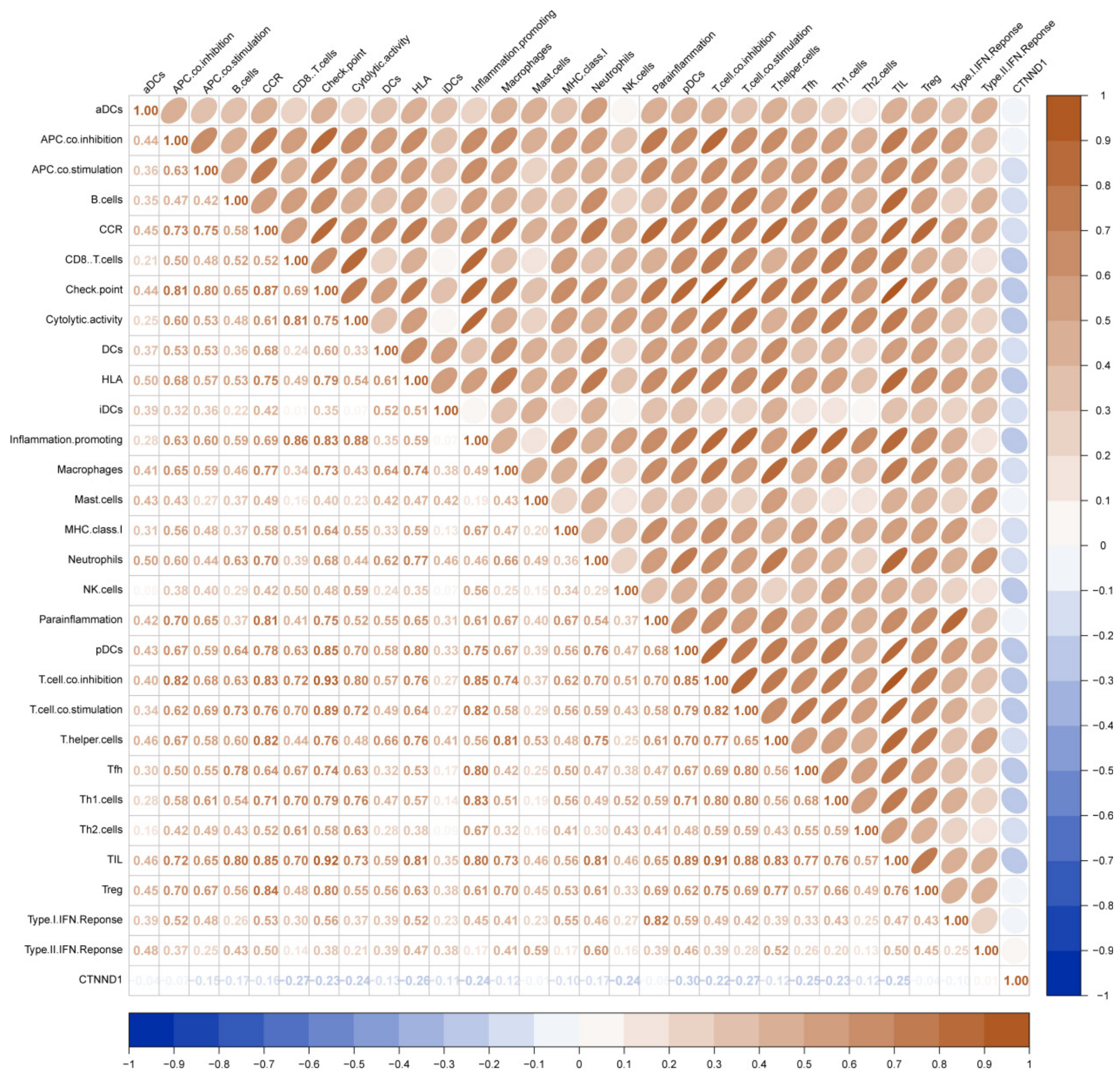

Supplementary Figure 7. Correlation between CTNND1 expression and the enrichment scores of immune subpopulations and immune-related signaling pathways in the TCGA cohort.

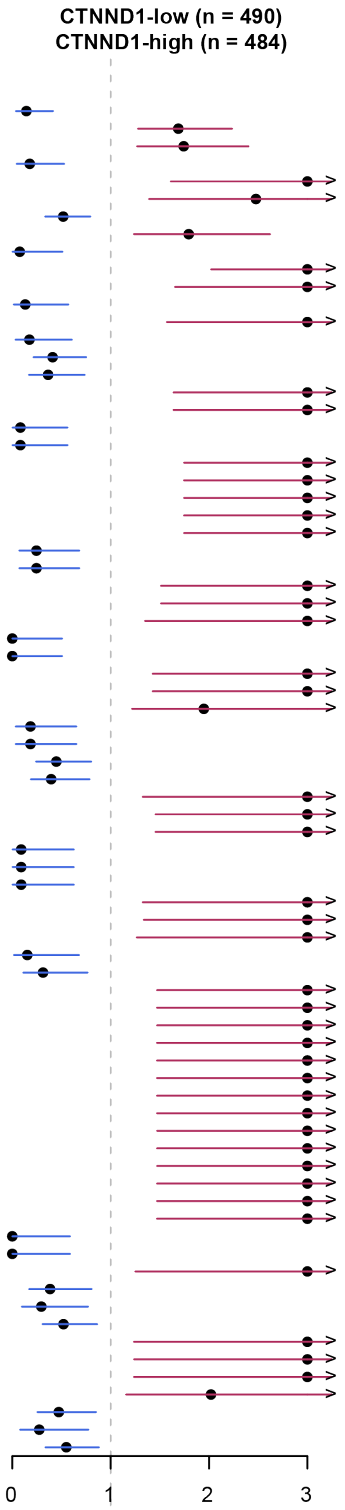

Gene	mutations in CTNND1-low	mutations in CTNND1-high	OR	P−value
ITGBL1	27	4	0.143	***
TP53	300	352	1.688	***
SYNE1	86	131	1.742	***
ST8SIA6	22	4	0.178	***
OR4K2	9	30	3.527	***
ZNF99	19	44	2.477	**
MXRA5	71	39	0.518	**
KMT2D	56	91	1.793	**
U2AF1	13	1	0.076	**
NMS	0	9	Inf	**
GRIPAP1	2	14	7.256	**
MCOLN3	15	2	0.132	**
GIGYF2	3	16	5.542	**
GALNT15	17	3	0.174	**
PCDHA2	40	17	0.41	**
OR4C16	32	12	0.364	**
ESCO1	1	11	11.35	**
MYOT	1	11	11.35	**
BUD13	12	1	0.083	**
TRBV19	12	1	0.083	**
HIST1H3F	0	8	Inf	**
LBR	0	8	Inf	**
MFGE8	0	8	Inf	**
PLBD2	0	8	Inf	**
ZSCAN32	0	8	Inf	**
DSC3	20	5	0.246	**
NEU4	20	5	0.246	**
C17orf104	2	13	6.724	**
SYT1	2	13	6.724	**
NUP210	8	24	3.14	**
CNTFR	9	0	0	**
PLCXD2	9	0	0	**
FBXO40	4	17	4.417	**
RASAL3	4	17	4.417	**
CDKN2A	32	58	1.947	**
LGI2	16	3	0.185	**
SLC35F3	16	3	0.185	**
TRPM6	41	19	0.448	**
NETO1	32	13	0.395	**
ZNF184	5	18	3.743	**
SAFB	1	10	10.298	**
SESN2	1	10	10.298	**
GJA9	11	1	0.09	**
PNPLA5	11	1	0.09	**
SLAMF8	11	1	0.09	**
OR52A1	4	16	4.148	**
L3MBTL4	3	14	4.828	**
ALG13	7	21	3.126	**
FAM73A	13	2	0.152	**
CCDC147	22	7	0.313	**
C1QTNF6	0	7	Inf	**
C9orf172	0	7	Inf	**
IGF2	0	7	Inf	**
KDM8	0	7	Inf	**
LNX2	0	7	Inf	**
LTV1	0	7	Inf	**
MLC1	0	7	Inf	**
MSL2	0	7	Inf	**
PCCB	0	7	Inf	**
POLDIP2	0	7	Inf	**
PRNT	0	7	Inf	**
SDR16C5	0	7	Inf	**
SLC15A4	0	7	Inf	**
TBCCD1	0	7	Inf	**
LMBR1L	8	0	0	**
ZWILCH	8	0	0	**
TRERF1	6	19	3.292	**
SLC9A2	28	11	0.384	**
LMO7	20	6	0.295	**
LRP1	50	27	0.52	**
OR52E8	5	17	3.527	**
SIDT1	5	17	3.527	**
ZNF28	5	17	3.527	**
TLN2	22	42	2.02	**
SIPA1L2	39	19	0.473	**
PSG4	18	5	0.274	**
CACNA1C	56	32	0.549	**

Supplementary Figure 8. Comparison of mutation variants between CTNND1-high and low groups.
